# Analysis of Low-Frequency 1/f Noise Characteristics for MoTe_2_ Ambipolar Field-Effect Transistors

**DOI:** 10.3390/nano12081325

**Published:** 2022-04-12

**Authors:** Bing Zhang, Congzhen Hu, Youze Xin, Yaoxin Li, Yiyun Xie, Qian Xing, Zhuoqi Guo, Zhongming Xue, Dan Li, Guohe Zhang, Li Geng, Zungui Ke, Chi Wang

**Affiliations:** 1 School of Microelectronics, Xi’an Jiaotong University, Xi’an 710049, China; hucongzhen@stu.xjtu.edu.cn (C.H.); youzxin@stu.xjtu.edu.cn (Y.X.); 1143506042@stu.xjtu.edu.cn (Y.L.); 4120105068@stu.xjtu.edu.cn (Y.X.); q.xing@stu.xjtu.edu.cn (Q.X.); guozhuoqi2004@163.com (Z.G.); lincolnxzm@163.com (Z.X.); dan.li@mail.xjtu.edu.cn (D.L.); zhangguohe@mail.xjtu.edu.cn (G.Z.); gengli@xjtu.edu.cn (L.G.); 2 Key Lab of Micro-Nano Electronics and System Integration of Xi’an City, Xi’an 710049, China; 3 Detector Laboratory of Southwest Institute of Technical Physics, Chengdu 610041, China; kezungui123@163.com; 4 ABAX Sensing Inc., Ningbo 315502, China; jxgx252584@163.com

**Keywords:** MoTe_2_, low-frequency noise, carrier number fluctuations, contact resistance

## Abstract

Low-frequency electronic noise is an important parameter used for the electronic and sensing applications of transistors. Here, we performed a systematic study on the low-frequency noise mechanism for both p-channel and n-channel MoTe_2_ field-effect transistors (FET) at different temperatures, finding that low-frequency noise for both p-type and n-type conduction in MoTe_2_ devices come from the variable range hopping (VRH) transport process where carrier number fluctuations (CNF) occur. This process results in the broad distribution of the waiting time of the carriers between successive hops, causing the noise to increase as the temperature decreases. Moreover, we found the noise magnitude for p-type MoTe_2_ FET hardly changed after exposure to the ambient conditions, whereas for n-FET, the magnitude increased by nearly one order. These noise characteristics may provide useful guidelines for developing high-performance electronics based on the emerging transition metal dichalcogenides.

## 1. Introduction

With the scaling of Si-based transistors approaching its limit, two-dimensional materials, as the possible building blocks of next generation electronics and optoelectronics, have attracted world-wide interest [1,2,3,4]. Although the zero-band gap of graphene greatly constrains its use in logic circuits, other 2D materials or derivatives, such as black phosphorus and transition-metal dichalcogenides (TMDs), show superior electrical and optoelectrical performance, and are considered to be promising candidates for the development of post-silicon devices [4,5,6,7,8,9,10,11,12]. 2H-type molybdenum ditelluride (MoTe_2_) is a layered compound with a band gap ranging from 0.81 eV (indirect) for bulk to 1.13 eV (direct) for monolayer, enabling its excellent performance in tunneling transistors, with high tunneling current and optoelectronic devices operating from the visible to the near-infrared range [11,13,14,15,16]. Moreover, due to its ambipolar transport property and its ability to improve mobility by tuning its carrier conduction type rationally, a variety of functional electronic devices can be realized, such as complementary metal-oxide-semiconductor transistors, logic inverters, and PN junctions, which paves the way for developing MoTe_2_-based high-performance electronics and optoelectronics [11,17,18,19,20].

Low-frequency noise usually manifests itself as slow fluctuations in the drain–source current, resulting from channel conductivity fluctuations [21,22,23,24]. It is a basic performance-limiting factor of electronic devices or circuits, in that the sensitivity and accuracy of electronic devices, such as sensors, amplifiers, or other digital and analog electronic devices, are ultimately defined by it [25,26,27,28]. With the decrease in circuit characteristic size, low-frequency noise becomes very important to the performance of the circuit [25,29,30,31,32,33,34,35]. Previous reports on low-frequency noise in graphene and MoS_2_ field-effect transistors suggest that the origin and mechanism of low-frequency noise is strongly dependent on the properties of the channel materials and device fabrication process [25,26,27,28,33,36,37,38]. To develop high-performance MoTe_2_-based electronic devices, a thorough characterization of specific features of low-frequency noise and the methods to reduce it is required.

In this work, we investigate the low-frequency noise in MoTe_2_ ambipolar field-effect transistors (FET). Noise measurement reveals that the noise mechanisms for both p-type and n-type conduction originate in CNF, causing the noise to increase as the temperature decreases. Subsequently, we find that large contact resistance at the metal–MoTe_2_ interface dominates the electrical and noise characteristics of the transistors in the “on” state. Finally, we investigate the impact of ambient conditions on the noise performance of MoTe_2_ n-FET and p-FET, finding that the noise in n-FET and p-FET changes variably following exposure to the atmospheric environment. The noise magnitude of n-FET increases by nearly one order, while in p-FET, little change is shown.

## 2. Materials and Methods

MoTe_2_ thin flakes were mechanically exfoliated using the Scotch tape method and transferred onto a degenerately n-doped Si/SiO_2_ (300 nm) substrate. The target MoTe_2_ thin flakes were identified by measuring the Raman scattering spectra with a 633 nm laser under an optical microscope, and the thickness of the flakes were determined by atomic force microscopy combined with the optical contrast. Standard electron-beam lithography was employed to define the electrical contact, and a metal stack of Cr/Au (5/60 nm) was deposited by electron beam evaporation. The electrical characterization of MoTe_2_ FET was performed in a lakeshore probe station (pressure lower than 10^−5^ Torr) using a Keithley 4200-SCS (Tektronix Inc., Beaverton, OR, USA) and a custom PDA fast probe noise measurement system.

## 3. Results

### 3.1. Basic Characteristics

**Figure 1:**
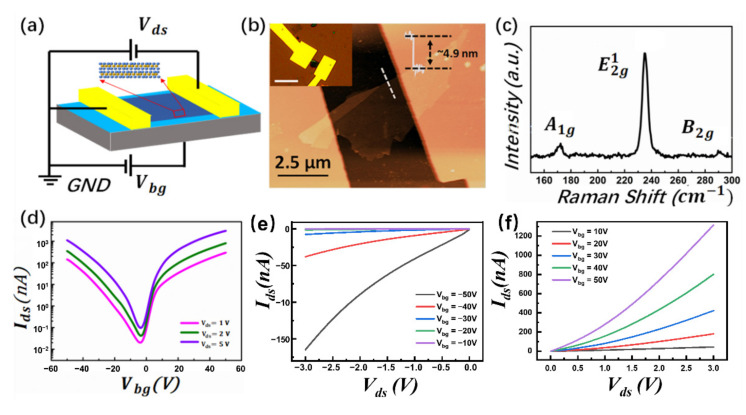
(**a**) Schematic image of MoTe_2_ transistor. (**b**) Atomic force microscope image of the transistor. The inset is the optical image of the transistor, scale bar is 5μm. (**c**) Raman spectrum of the MoTe_2_ measured under 633 nm laser flake in (**b**). (**d**) Transfer curves of the MoTe_2_ transistor with the back gate voltage sweeping from −50 to 50 V at a step of 0.8 V at different bias voltage: *V_ds_* = 1.0 V, 2.0 V, and 5.0 V, respectively. (**e**) Output curves of the transistor with bias voltage swept from −3.0 V to 0 V under different back gate voltages ranging from −50 to −10 V at a step of 10 V. (**f**) Output curves of the transistor with bias voltage swept from 0 to 3 V under different back gate voltages ranging from 10 to 50 V at a step of 10 V.

A schematic image of the MoTe_2_ field-effect transistor is shown in Figure 1a. The thickness of the flake was confirmed to be 4.9 nm by a surface line profile, as shown in Figure 1b, and the inset is an optical image of the device. Figure 1c is the Raman spectrum collected from the position indicated in Figure 1b. The peaks at around 171, 234, and 289 cm^−1^ can be attributed to the A*_1g_*, E*^1^_2g_*, and B*^1^_2g_* vibration modes, respectively, which is consistent with previous reports [13,14,15]. The room-temperature transfer characteristics of the device under different bias voltages are shown in Figure 1d. The increase in the drain current I_ds_ with increasing gate voltage for both negative and positive polarities demonstrates the ambipolar operation of the MoTe_2_ FET, and the obvious saturation trend of the drain current at positive gate voltage (~7.5 V) can be a result of the contact resistance at the metal–MoTe_2_ interface [39]. The on/off ratio at *V_ds_* = 1.0 V is found to be ~7.1 × 10^3^ for MoTe_2_ p-FET, and ~1.5 × 10^4^ for MoTe_2_ n-FET, respectively. Field-effect mobility can be extracted from the equation:

(1) $$\mu_{FE}=\frac{1}{C_{ox}}\frac{1}{V_{ds}}\frac{L}{W}\frac{dI_{ds}}{dV_{bg}}$$

In this equation, L and W are the length and width of the channel, respectively. *C_ox_* is the gate oxide capacitance per unit area, and *V_ds_* is the source–drain bias voltage. The linear fitting was taken at the linear region of the transfer characteristics, and a mobility of 2.66 cm^2^/Vs for hole and 2.03 cm^2^/Vs for electron can be extracted, respectively. Figure 1e,f reveal the output characteristics of the MoTe_2_ FET with a bias voltage ranging from −3 to 0 V for p-FET, and 0–3 V for n-FET at different gate voltages. The good linear shape at small bias voltage and the obvious saturation of the current for p-FET imply that the contact between MoTe_2_ and metal electrode is ohmic or the contact resistance is comparably small. However, for n-FET, the drain current increases nonlinearly with bias voltage, indicating the existence of significant contact resistance at the metal–MoTe_2_ interface, which will suppress the electron injection from the metal electrode into the conducting channel and may cause series resistance noise [40].

### 3.2. Noise Characteristics

**Figure 2:**
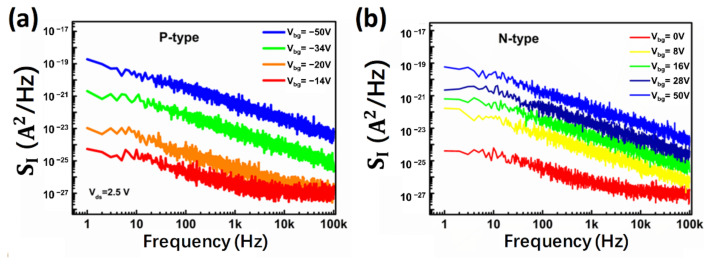
(**a**) Noise power spectral density *S_I_* as a function of frequency at different gate voltages for MoTe_2_ p-FET. (**b**) Noise power spectral density *S_I_* as a function of frequency at different gate voltages for MoTe_2_ n-FET.

The noise characterization of MoTe_2_ FET is shown in Figure 2. The noise power spectral density *S_I_* of the MoTe_2_ p-FET at four different gate voltages between 1 Hz to 100 kHz with a bias voltage kept at *V_ds_* = 2.5 V is shown in Figure 2a. When the p-FET is in the “on” state, the noise spectra follow the typical 1/f dependence and can be quantitatively characterized by

(2) $$S_{I}=AI_{ds}^{2}/f^{\beta }$$

In this equation, *S_I_* is the current noise power spectral density, *A* is the noise amplitude, *I_ds_* is the current through the device channel, f is the frequency, and *β* is the frequency exponent. However, when the gate voltage is close to the flat band voltage, or the device is in the “off” state, the noise spectra at a high frequency increase and deviate from 1/f dependence because of the instrumental noise floor. Figure 2b shows the noise characteristics of the MoTe_2_ n-FET in the same device and similar behavior can be observed.

**Figure 3:**
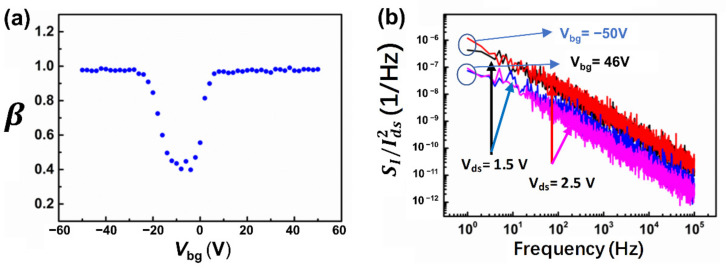
(**a**) Frequency exponent β as a function of gate voltage. (**b**) Normalized noise spectra $S_{I}/I_{ds}^{2}$ as a function of frequency at different bias voltage: *V_ds_* = 1.5 V, 2.5 V under different gate voltage: *V_bg_* = −50 V, 46 V.

The extracted frequency exponent *β* as a function of *V_bg_* was extracted from a least-square fit of Figure 2a using Equation (2), which ranges from 0.95 to 1.1 in the “on” state as shown in Figure 3a. To determine whether the metal–MoTe_2_ contact barrier contributes to the noise of the MoTe_2_ FET, the normalized noise power spectra $S_{I}/I_{ds}^{2}$ of both p-FET (*V_bg_* = −50 V) and n-FET (*V_bg_* = 46 V), as a function of frequency at different bias voltages, were performed, as shown in Figure 3b. It was found that the normalized noise power spectra $S_{I}/I_{ds}^{2}$ for p-FET are independent of the bias voltage, indicating that the noise mainly originated from the MoTe_2_ conducting channel itself. However, for n-FET, the normalized noise power spectra $S_{I}/I_{ds}^{2}$ are strongly dependent on bias voltage, suggesting the contact barrier is one of the main contributors to the noise of n-FET. These results are consistent with initial DC characterization results that significant contact resistance exists at the metal–MoTe_2_ interface.

There are two models to describe the noise mechanism of the conventional FET: the carrier number fluctuation (CNF) model, which can be expressed by:

(3) $$S_{I}/I_{ds}^{2}=\left(g_{m}/I_{ds}\right)2S_{Vfb}$$

(4) $$S_{Vfb}=q^{2}K_{B}TN_{it}/\mathrm{WL}C_{ox}{}^{2}f$$

where *S_vfb_* is the flat-band voltage spectral density and *q* is the elementary charge, *K_B_* is the Boltzmann constant, *T* is the absolute temperature, *N_it_* is the effective trap density, and *C_ox_* is the gate unit capacitance, respectively. The other model is the Hooge mobility fluctuation (HMF) model which is expressed by

(5) $$S_{I}/I_{ds}^{2}=q\alpha_{H}\mu_{eff}V_{ds}/fL^{2}I_{ds}$$

where the Hooge parameter *α_H_* is an empirical dimensionless constant. If the series resistance contributes to the low-frequency noise, the total current noise changes into the form

(6) $$S_{I}/I_{ds}^{2}={\left(S_{I}/I_{ds}^{2}\right)}_{channel}+{\left(I_{ds}/V_{ds}\right)}^{2}S_{Rsd}$$

where *S_Rsd_* is the spectral density of series resistance [22,23]. From the equation, we can see that if the normalized drain current noise increases at a high current, it can be indicative of an enhanced low-frequency noise contribution of the series resistance.

**Figure 4:**
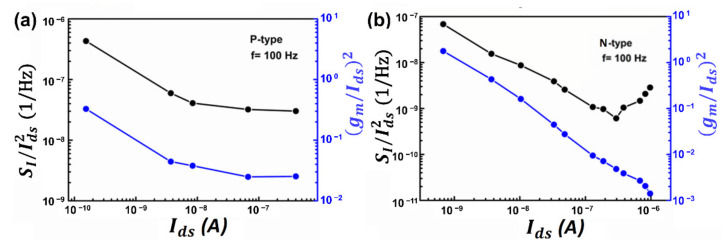
(**a**) $S_{I}/I_{ds}^{2}$ and its corresponding ${\left(g_{m}/I_{ds}\right)}^{2}$ as a function of drain current for MoTe_2_ p-FET. (**b**) $S_{I}/I_{ds}^{2}$ and its corresponding ${\left(g_{m}/I_{ds}\right)}^{2}$ as a function of drain current for MoTe_2_ n-FET.

To uncover the noise mechanism of this few-layer MoTe_2_-based transistor, $S_{I}/I_{ds}^{2}$ at *f* = 100 Hz and its corresponding ${\left(g_{m}/I_{ds}\right)}^{2}$ at 300 K as a function of the drain current for the MoTe_2_ p-FET and n-FET are plotted in Figure 4a,b, respectively. From Figure 4a, we can observe that $S_{I}/I_{ds}^{2}$ and its corresponding ${\left(g_{m}/I_{ds}\right)}^{2}$ follow the same trend over a wide drain current range, indicating that the low-frequency noise in MoTe_2_ p-FET comes from the carrier number fluctuation [24]. However, for n-FET, as shown in Figure 4b, we found that $S_{I}/I_{ds}^{2}$ and its corresponding ${\left(g_{m}/I_{ds}\right)}^{2}$ follow the same trend. At small currents larger than 2.0 × 10^−7^ A, the normalized noise power spectra $S_{I}/I_{ds}^{2}$ deviate from their corresponding ${\left(g_{m}/I_{ds}\right)}^{2}$ and start to increase alongside the drain current. Therefore, when the drain current is *I_ds_* < 2.0 × 10^−7^ A, noise is from the carrier number fluctuation, while when the drain current is *I_ds_* > 2.0 × 10^−7^ A, the noise of the n-FET mainly comes from the metal–MoTe_2_ contact barrier, which is consistent with results shown in Figure 3b.

**Figure 5:**
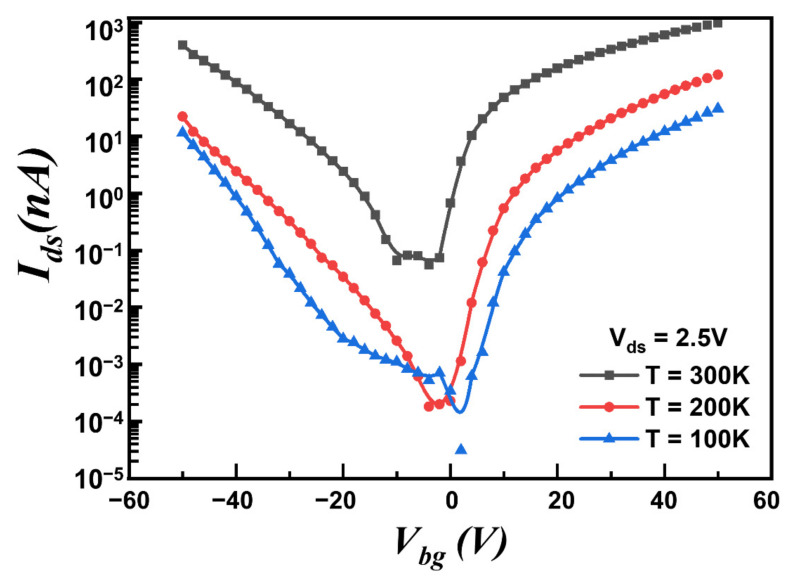
Transfer curves of the MoTe_2_ transistor at different temperatures from 100 to 300 K at a step of 100 K.

**Figure 6:**
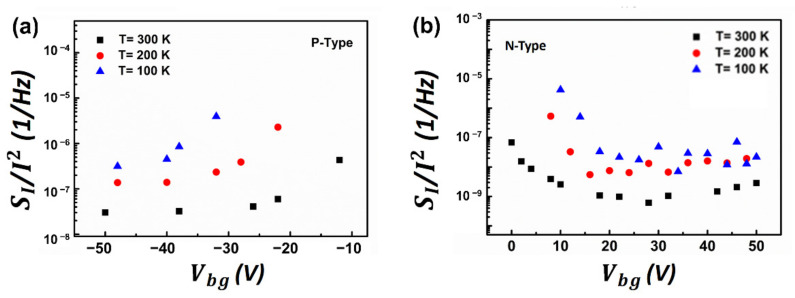
(**a**) $S_{I}/I_{ds}^{2}$ as function of gate voltage for p-FET and (**b**) for n-FET at different temperature from 100 to 300 K.

To further investigate the noise mechanisms, we characterized the transfer characteristics of the device (Figure 5), and normalized the noise power spectra $S_{I}/I_{ds}^{2}$ as a function of gate voltage (Figure 6a for p-FET and Figure 6b for n-FET, respectively) at different temperatures from 100 to 300 K. At different temperatures, $S_{I}/I_{ds}^{2}$ almost follows the same trend for both p-FET and n-FET, indicating that the noise mechanism is independent of temperature. Moreover, we observed that the magnitude of normalized noise spectra $S_{I}/I_{ds}^{2}$ for both MoTe_2_ p-FET and n-FET decreases as temperatures increase, which is inconsistent with previous reports which suggest that the magnitude of noise amplitude decreases alongside the temperature when dominated by a thermally activated process [32]. Previous theoretical studies reported that the variable range hopping (VRH) transport in transistors will result in the broad distribution of the waiting time of the carriers between successive hops, causing the noise to increase as the temperature decreases [41,42].

**Figure 7:**
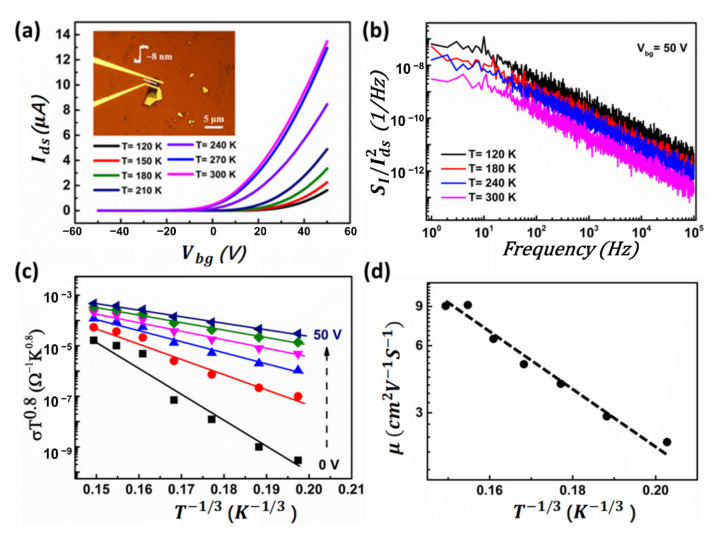
Conduction mechanism in an MoTe_2_ transistor. (**a**) Transfer curves of MoTe_2_ FET at different temperatures from 120 to 300 K at a step of 30 K. (**b**) Normalized noise spectra $S_{I}/I_{ds}^{2}$ as a function of frequency at different temperatures from 120 s to 300 K at a step of 60 K. (**c**) Conductivity σ as a function of temperature at different gate voltages ranging from 0 to 50 V at a step of 10 V. (**d**) Field-effect mobility of the MoTe_2_ transistor as a function of temperature.

**Figure 8:**
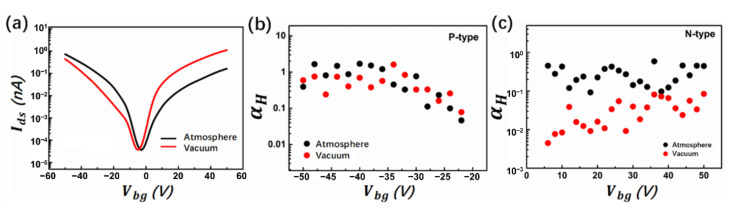
Impact of ambient conditions on the noise of MoTe_2_ transistor. (**a**) Transfer curves of MoTe_2_ FET before and after exposure to ambient conditions. (**b**) Hooge parameter $\alpha_{H}$ as a function of gate voltage before and after exposure to ambient conditions for MoTe_2_ p-FET and (**c**) for MoTe_2_ n-FET.

To experimentally validate the theoretical predictions, and demonstrate whether this phenomenon is occasional, several MoTe_2_ devices with a channel thickness ranging from 4.9 nm to 12 nm were fabricated and studied experimentally. The noise characteristics were electrically measured at different temperatures. All these experiments show the same pattern around noise characteristics. Experimental results of MoTe_2_ devices with a channel thickness of 8.0 nm are shown in Figure 7a,b. The same decreasing behavior of $S_{I}/I_{ds}^{2}$ with increasing temperatures was also observed. Therefore, conductivity (σ) of two terminals as a function of temperature was characterized to determine the conduction mechanism within the MoTe_2_ channel, as shown in Figure 7c. We found that the variation of σ with T in MoTe_2_ transistors can be modeled well with the variable *I_ds_* hopping (VRH) transport.

(7) $$\sigma =AT^{-0.8}exp\left[{\left(T_{0}/T\right)}^{1/3}\right]$$

(8) $$N=(V_{bg}-V_{th})LWC_{ox}/q$$

where $V_{th}$ is the threshold voltage of the device, is the total number of carriers in the conducting channel, before and after the exposures were shown in Figure 8b for p-FET and Figure 8c for n-FET, respectively. As demonstrated, for n-FET, the magnitude of noise changes increases nearly by one order, while for p-FET, it shows no apparent change, which agrees with previous studies [25,43,44,45]. For n-FET, adsorbates from ambient conditions function as trapping centers, trapping and scattering the electron within the channel, while for p-FET, the concentration of carriers will increase because of the ambient doping; thus, conductance increases and noise, in comparison, either decreases or hardly changes.

## 4. Conclusions

In conclusion, we characterized the noise features in MoTe_2_ few-layered ambipolar transistors. The noise mechanism of the MoTe_2_ p-FET and n-FET was characterized as carrier number fluctuations, which were caused by variable range hopping transport rather than thermally activated transport in the MoTe_2_ conducting channel, and stayed the same with temperature. The noise of n-channel and p-channel of MoTe_2_ ambipolar transistors showed a different response upon exposure to ambient conditions, which indicates that n-channel transport is more sensitive to ambient conditions. These noise characteristics may provide useful guidelines to develop high-performance electronics based on the emerging transition metal dichalcogenides and can be a useful diagnostic tool to identify the conduction mechanism.

## Data Availability

Not applicable.
